# Clinical Characteristics of Eyes with Neovascular Age-Related Macular Degeneration and Retinal Pigment Epithelium Tears

**DOI:** 10.3390/jcm12175496

**Published:** 2023-08-24

**Authors:** Junya Nagata, Satomi Shiose, Keijiro Ishikawa, Takuma Fukui, Kumiko Kano, Kenichiro Mori, Takahito Nakama, Shoji Notomi, Koh-Hei Sonoda

**Affiliations:** Department of Ophthalmology, Graduate School of Medical Sciences, Kyushu University, Fukuoka 812-8582, Japan

**Keywords:** age-related macular degeneration, anti-vascular endothelial growth factor therapy, cleft, retinal pigment epithelium tear, subretinal hyporeflective space

## Abstract

Background: Although anti-vascular endothelial growth factor (anti-VEGF) therapy is the first choice of treatment for eyes with neovascular age-related macular degeneration (AMD), it sometimes results in retinal pigment epithelium (RPE) tears. This study presents the detailed clinical characteristics of RPE tears to help predict their occurrence before anti-VEGF therapy initiation. Methods: This study retrospectively analyzed neovascular age-related macular degeneration (nAMD) patients who visited the Kyushu University Hospital and started anti-VEGF therapy between April 2013 and June 2020. Using medical records, we collected the clinical data of patients with RPE tears, including age, sex, best-corrected visual acuity (BCVA), number of anti-VEGF drug injections and the type and size of pigment epithelial detachment (PED). Results: RPE tears occurred in 16 (1.50%) eyes of 16 patients in all 1068 nAMD eyes of 987 patients. The mean age of these patients with RPE tear was 81.7 ± 8.7 years. Fifteen eyes had typical AMD and one eye had polypoidal choroidal vasculopathy. The mean number of anti-VEGF drug injections before RPE tears was 5.0 ± 5.1. All patients experienced PED before the RPE tear (hemorrhagic, 4 eyes; serous vascular, 2 eyes; fibrovascular, 10 eyes). The average PED height and area were 615.7 ± 175.3 μm and 21.0 ± 7.2 mm^2^, respectively. The sub-RPE cleft was observed in 10 eyes. The logMAR BCVA immediately after the RPE tear (0.73 ± 0.40) at 6 months (0.86 ± 0.51) and 12 months (0.84 ± 0.43) after the RPE tear were significantly worse than that before the RPE tear (0.58 ± 0.31; *p* < 0.05). The BCVA of patients with RPE tears that spread to the fovea was poorer than that of patients without RPE tears. Conclusions: In patients with nAMD, RPE tears tended to occur in typical AMD eyes with high or large PEDs, and sub-RPE clefts. The visual prognosis depended on whether the RPE tear included the fovea.

## 1. Introduction

Neovascular age-related macular degeneration (nAMD) is a disease that causes chronic visual impairment attributable to damage resulting from exudation and hemorrhage associated with choroidal neovascularization (CNV) in elderly individuals. Although the intravitreal injection of anti-vascular endothelial growth factor (VEGF) treatment is the first choice of therapy, its associated complications are problematic. One serious complication is a retinal pigment epithelium (RPE) tear; however, its main pathogenic mechanism is currently unknown. RPE tears can occur spontaneously in eyes with nAMD. In such cases, leakage from the occult CNV membrane could sufficiently increase hydrostatic pressure, resulting in the breakdown of intercellular adhesion between the RPE and Bruch membrane [1]. Intravitreal administration of anti-VEGF treatment is thought to cause contraction of the CNV membrane attached to the RPE, resulting in excessive tension on the RPE and development of the RPE tear [2].

Large pigment epithelial detachment (PED) basal diameters, high PED, short PED durations, serous vascular PED, radial hyperreflective lines of PED lesions, and small CNV/PED ratios are predictors of and risk factors for RPE tears [3]. Although the prediction of the onset of this condition is necessary, there have been few reports of and clear opinions regarding the risks of RPE tears because they do not occur frequently. The accumulation of more reports would allow a better understanding of the pathogenesis of RPE tears. Therefore, this study aimed to report the clinical features of eyes with nAMD and RPE tears.

## 2. Methods

This retrospective study used the medical records of nAMD patients who started 3-monthly intravitreal ranibizumab (0.5 mg/0.05 mL) or aflibercept (2 mg/0.05 mL) injections as initial treatment at Kyushu University Hospital from April 2013 to June 2020. Additional injections were administered using a treat-and-extend regimen at each follow-up visit. All patients underwent complete ophthalmic examinations, including color fundus photography, optical coherence tomography (OCT) (SPECTRALIS; Heidelberg Engineering, Heidelberg, Germany), fluorescein angiography, indocyanine green angiography, and fundus autofluorescence imaging (SPECTRALIS; Heidelberg Engineering) before anti-VEGF treatment initiation. Consecutive patients who developed RPE tears after intravitreal anti-VEGF injections and were followed-up for more than 12 months were selected for this study. Written informed consent was obtained from all patients with age-related macular degeneration (AMD). This study was approved by the Institutional Review Board of Kyushu University Hospital and was conducted in accordance with the Declaration of Helsinki for research involving human subjects (approval number: 2019-630).

Using the medical records, we collected the following data of patients with RPE tears: age, sex, medical history, best-corrected visual acuity (BCVA), and number of anti-VEGF drug injections. The BCVA was converted from the decimal BCVA to the logarithm of the minimum angle of resolution (logMAR) and compared before, immediately after, and 12 months after the onset of the RPE tear.

The CNV membrane location and AMD classification, including typical AMD (tAMD), polypoidal choroidal vasculopathy (PCV), and retinal angiomatous proliferation (RAP), were determined using OCT, fluorescein angiography, and indocyanine green angiography images. All B scan images taken in 6 mm × 6 mm volume scan mode of OCT were checked. The PED height was measured using OCT as the vertical distance between Bruch’s membrane and the maximum PED apex. The PED area was determined based on the extent of PED exhibited by the fluorescein angiography images. PED was classified into the following three types based on fundus photographs and OCT images: hemorrhagic PED, serous vascular PED, and fibrovascular PED. RPE tear was classified according to the RPE grading scheme per Sarraf et al. [4]. The paired t test was used to determine changes in the logMAR visual acuity; *p* < 0.05 was considered significantly different.

## 3. Results

Of the 1068 treatment-naïve nAMD eyes included in this study (987 patients; mean observation period, 36.4 months), 16 (1.5%) eyes of 16 patients developed RPE tears (Table 1 and Table 2). These tears occurred in 10 male and 6 female patients (age range, 64–97 years; mean age, 81.7 years; standard deviation [SD], ±8.7 years).

**Figure 1:**
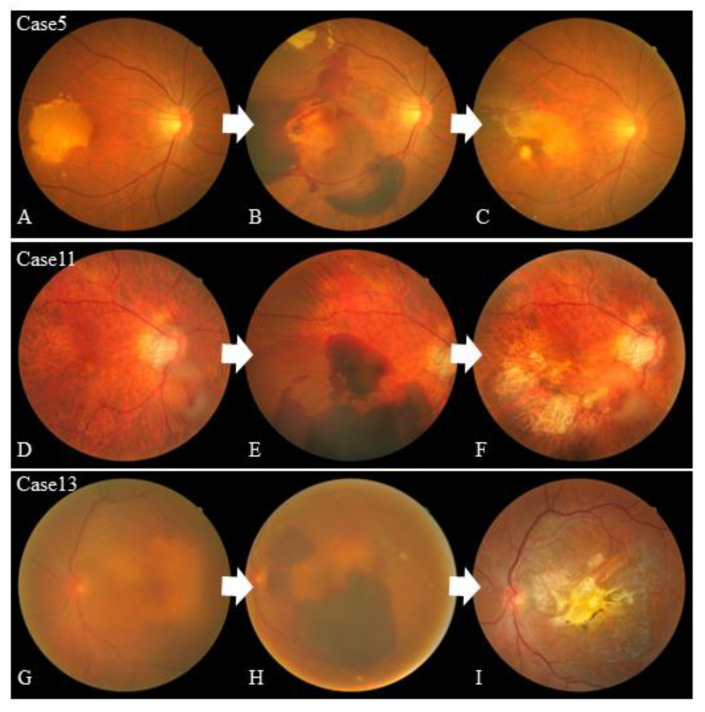
Color fundus photographs of cases 5, 11, and 13. The color fundus photographs of cases 5, 11, and 13 before the retinal pigment epithelium (RPE) tear (**A**,**D**,**G**), after the onset of massive rapid hemorrhage retention (**B**,**E**,**H**), and after hemorrhage resolution of the RPE tear (**C**,**F**,**I**). Cases 5 and 13 (**A**,**G**) included fibrovascular pigment epithelial detachment (PED) managed by treat-and-extend anti-vascular endothelial growth factor (anti-VEGF) therapy before the RPE tear. Case 5 included a large subretinal RPE hemorrhage soon after the anticoagulant was started. Macular polyps in case 11 were controlled with treat-and-extend anti-VEGF therapy, but rapid bleeding from an extramacular polyp caused a large RPE tear.

Regarding systemic factors, 3 (18.8%) patients had diabetes mellitus, 13 (81.2%) patients had hypertension, 6 (37.5%) patients had a history of smoking, and 6 (37.5%) patients used oral anticoagulants. An RPE tear with a large subretinal hemorrhage developed in one patient soon after an anticoagulant was administered (Figure 1, case 5).

The disease types of eyes with RPE tear were tAMD in 15 (93.8%) eyes and PCV in one eye. Fluorescein angiography revealed that all eyes had occult type CNV.

**Figure 2:**
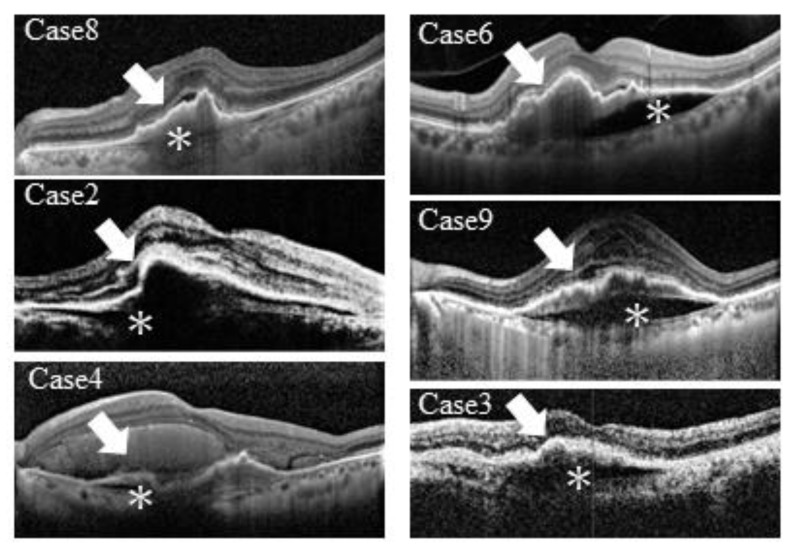
Spectral-domain optical coherence tomography images before the retinal pigment epithelium tear. The optical coherence tomography (OCT) images of cases 2, 3, 4, 6, 8, and 9 before the onset of the RPE tear show a jagged surface (arrow), indicating that the choroidal neovascular membrane (CNVM) is adhered to the bottom of the retinal pigment epithelium (RPE) and hyporeflective spaces under the RPE (*). The coexistence of these two findings may be a significant risk factor for the development of RPE tears in eyes with fibrovascular pigment epithelial detachment (PED).

All eyes had PED before the RPE tear (hemorrhagic PED, 4 eyes; serous vascular PED, 2 eyes; fibrovascular PED, 10 eyes). The mean height of PED was 615.7 μm (SD, ±175.3 μm). In 11 (68.8%) eyes, the height of PED was more than 550 μm. The average area of PED that could be measured before the RPE tear was 21.0 mm^2^ (SD, ±7.2 mm^2^). The hyporeflective space (cleft) (Figure 2) and contractile folds indicating that the CNV membrane was adhered to the bottom of the RPE were observed in 10 of 16 eyes.

One patient spontaneously developed an RPE tear just before anti-VEGF therapy was started. Fifteen patients developed RPE tears after anti-VEGF was started. Aflibercept and ranibizumab were the anti-VEGF drugs used immediately before the RPE tear for 10 eyes and for 4 eyes, respectively. After switching from ranibizumab to aflibercept, an RPE tear occurred in one eye. RPE tears developed in four eyes after the first anti-VEGF drug injection. The mean number of anti-VEGF drug injections administered before the RPE tear was 5.0 (SD, ±5.1).

**Figure 3:**
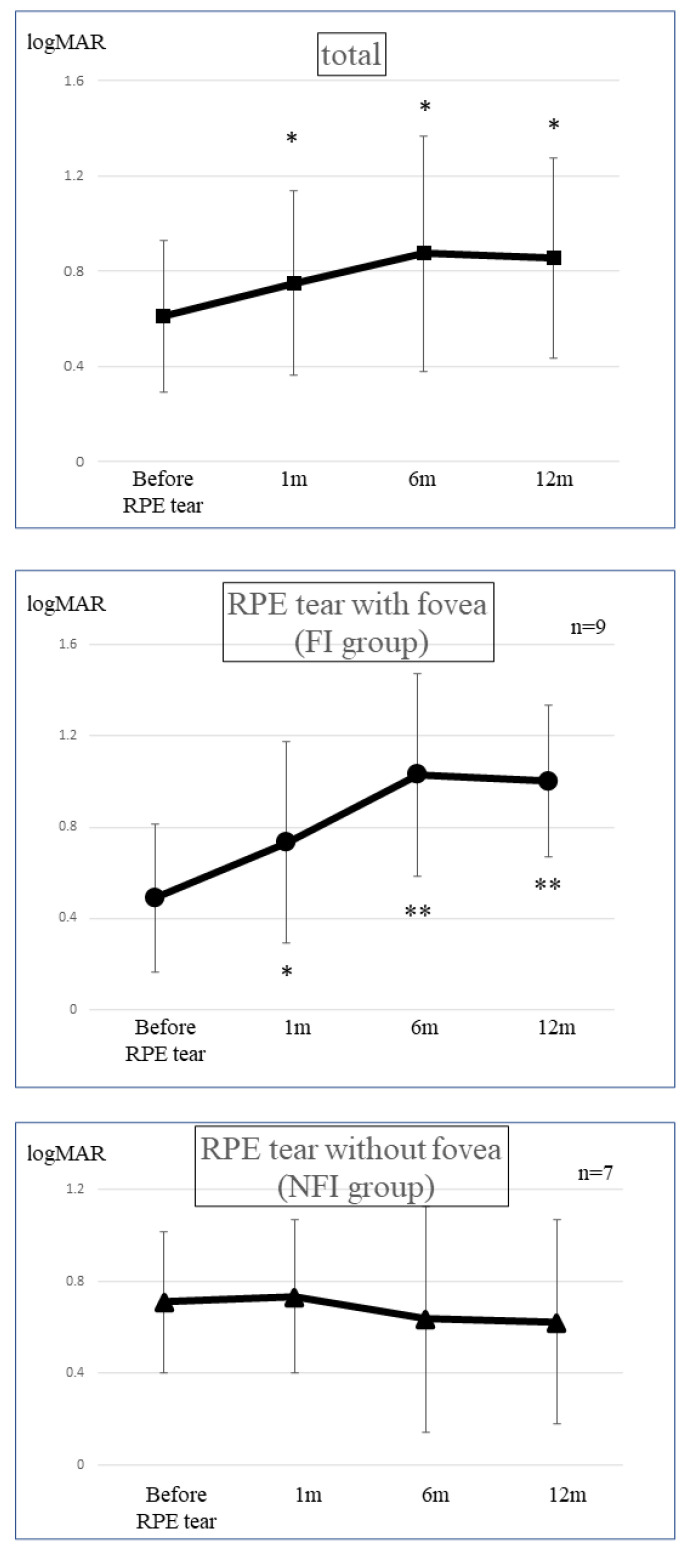
Best-corrected visual acuity changes after retinal pigment epithelium tear. The mean logMAR best-corrected visual acuity (BCVA) before and 1 month, 6 months, and 12 months after the retinal pigment epithelium (RPE) tear are shown. The mean BCVA of all eyes at 1 month after the RPE tear was significantly worse than that before the tear and remained worse until 12 months thereafter (paired *t*-test; * *p* < 0.05) (**top**). The mean BCVA of the eyes with RPE tears with foveal involvement (*n* = 9) was significantly worse during follow-up (paired *t* test; * *p* < 0.05; ** *p* < 0.01) (**middle**). However, the BCVA of eyes with RPE tears without foveal involvement (*n* = 7) did not worsen after treatment throughout the follow-up period (**bottom**).

In the group of eyes with RPE tears with fovea involvement (FI group; *n* = 9 eyes), the logMAR visual acuity 12 months after the RPE tear was 1.00 (SD, ±0.35), which was significantly worse than that before the RPE tear (0.49; SD, ±0.30; *p* = 0.008). However, in the group of eyes with RPE tears without fovea involvement (NFI group; *n* = 7 eyes), the logMAR visual acuity before onset and 12 months after onset were 0.71 (SD, ±0.31) and 0.62 (SD, ±0.44), respectively, indicating no trend of deterioration (*p* = 0.436) (Figure 3). Although there was no significant difference in visual acuity before the RPE tear between the FI and NFI groups, the visual acuity of the NFI group was significantly better than that of the FI group at 12 months after the RPE tear (*p* < 0.05) (Figure 3). No other complications, such as endophthalmitis or systemic adverse events, were observed.

Some PED areas were unmeasurable, because not all of the PEDs were within the photographing range.

## 4. Discussion

An RPE tear is a serious complication of nAMD that is irreversible and has no effective treatment. When it spreads to the fovea, vision is rapidly reduced. RPE tears naturally occur in approximately 10% of AMD eyes with PED caused by a rapid increase in hydrostatic pressure and fluid retention because of CNV activity [1]. Of the cases reported here, one eye spontaneously developed an RPE tear before the start of anti-VEGF treatment because of the acute increase in CNV activity. Although there is no solid evidence indicating that anti-VEGF treatment triggers RPE tears, it has been reported that their incidence increased after the introduction of anti-VEGF drugs. It is speculated that this occurs because the contraction of CNV membrane after anti-VEGF treatment could create acute tractional force in the RPE layer [2].

The incidence rate of RPE tears in our study was 1.5% (of the total number of eyes with nAMD). Data of phase III clinical trials of anti-VEGF drugs, such as MARINA, CATT, and VIEW, showed a very low incidence rate of RPE tears (0–0.3%) [3]. However, this may have occurred because eyes at risk for RPE tears were excluded from these studies. Other reports of real-world clinical studies described incidence rates of 14% to 17% among vascular PED cases [5,6], which are higher than the 10% incidence rate of spontaneous RPE tears with vascular PED. This may indicate that anti-VEGF therapy increases the incidence of RPE tears. Our overall incidence of RPE tears with nAMD of 1.59% is comparable to those reported by Chan et al. [7] (2.2%) and Ronan et al. [8] (0.8%).

Regarding systemic factors, 81.2% of patients with RPE tears had hypertension. This is a predictable result. Hypertension can exacerbate the pathogenesis of AMD because more anti-VEGF injections are sometimes necessary for patients with both AMD and hypertension [9]. Additionally, in CNV membrane with fragile vessel walls, fluctuations in blood pressure can cause a rapid increase in exudation and hemorrhage, which may lead to a rapid increase in hydrostatic pressure associated with PED and a higher incidence of RPE tears. The prevalence of hypertension in Japan is 34% of the total, and 50% at age 40~74, the age of onset of AMD, and about 72% at age 75 or older [10]. Although the incidence of HT increases with age, it could be said that 81.2% is higher than percentage of patients with hypertension in Japan.

The disease type of all eyes with RPE tears were tAMD or PCV with occult CNV membranes. No eyes with RAP experienced RPE tears during our observation period. AMD types associated with the risk of RPE tears have been reported. Cho et al. [11]. reported that tAMD and RAP are associated with similar incidence rates of RPE tears (10.7% and 9.8%, respectively); furthermore, their observed rates were significantly higher than that associated with PCV. They speculated that because of the lack of strong adhesion between polypoidal lesions and RPE layers, polyp regression after intravitreal anti-VEGF injections might not result in sufficient contractile force to allow RPE tear formation, and that the risk of RPE tears in eyes with PCV might be relatively lower than that for eyes with tAMD or RAP. Sarraf et al. [5] reported that three of seven eyes with RAP experienced RPE tears within 12 months during their prospective study involving patients with AMD and vascular PED. Although there have been few reports of RPE tears in eyes with PCV, our patient with PCV experienced rapid and large subretinal and sub-RPE hemorrhage, which led to an RPE tear. Therefore, it is necessary to consider the possibility of RPE tears in eyes with PCV as well.

Regarding the type of anti-VEGF agent, 10 of the 16 patients developed RPE tears after the administration of aflibercept or after switching from ranibizumab to aflibercept. Because there have been a previous report of higher RPE tear incidences with higher concentrations of drugs [5], when using more potent agents, attention should be focused on the occurrence of RPE tears.

Regarding the timing of RPE tear development after the introduction of anti-VEGF drugs, many reports indicated that these tears are more likely to occur after the first three anti-VEGF injections [5,7]. In our study, 53% of eyes experienced RPE tears during the loading phase of anti-VEGF drug therapy. However, all other eyes in which RPE tears occurred after 5 to 18 anti-VEGF drug injections experienced a rapid increase in bleeding or CNV activity because of undertreatment. An optimal treatment plan and the prevention of bleeding may reduce the incidence of RPE tears.

All RPE tear cases included in this observation series were complicated with hemorrhagic, fibrovascular, or serous vascular PED before the onset of the RPE tear. Although a previous report has identified serous vascular PED and fibrovascular PED as risk factors for RPE tears [3], our results suggest that all types of PED are susceptible to RPE tears and require attention. The mean PED length and area of the observed cases were 615.7 μm (SD, ±175.3 μm) and 21.0 mm^2^ (SD, ±7.2 mm^2^), respectively. These results are similar to those of studies [5,8] that found a higher risk of RPE tears with higher PED heights (>550 μm) or larger PED areas (diameter > 5000 μm). In the present study, five (29%) patients with PED heights less than 550 μm also developed RPE tears, and three of these five patients experienced extensive subretinal hemorrhage immediately before the onset of the tear. In such cases, the massive hemorrhage caused by the activation of CNV may have rapidly increased the volume of PED, which caused RPE tears and subretinal hemorrhage.

Hyporeflective spaces (cleft) and contractile folds indicating that the CNV membrane was under the RPE layer were observed in 10 of the 16 eyes. Even in eyes at seemingly low risk for low PED heights, RPE tears occurred in those with clefts and jagged CNV membrane surfaces. A previous study has reported that this hyporeflective band is often associated with the outcomes of CNV membrane contractions [12]. Moreover, Mukai et al. [13] showed that RPE tears developed in 23% of eyes with this cleft. The coexistence of these two findings (cleft and jagged CNV membrane surface) with fibrovascular PED may be an important predictor of RPE tear development. Therefore, clinicians should check all slices of the OCT volume scans of the 6 × 6-mm area to identify these findings.

At 12 months after the onset of RPE tears, the FI group tended to exhibit a greater degree of visual acuity loss than the NFI group. Currently, there is no effective treatment for RPE tears; furthermore, when these tears involve the fovea, the visual acuity prognosis is poor. For eyes with large PED and high PED, treatment should be initiated as soon as possible before spreading to the fovea occurs. When PED already involves the fovea and the CNV activity is high, it is necessary to fully explain the risk of visual acuity loss to patients before treatment.

This study had some limitations. This study included only 16 eyes. Furthermore, no comparative study of each clinical characteristic was conducted between groups with and without RPE tears. Additionally, it involved a single center and similar racial and ethnic groups. However, the current data regarding RPE tears are insufficient. Therefore, we believe that these results provide helpful information.

## 5. Conclusions

This observational case series indicated that hypertension, large PED, high PED, and sub-RPE clefts with jagged CNV membrane surfaces with fibrovascular PED could be important clinical features of RPE tear development in eyes with AMD.

## Figures and Tables

**Table 1:** Clinical characteristics of 16 patients who developed RPE tears after intravitreal anti-VEGF drug injections for nAMD.

	Age (years)	Sex	Affected Eye	Hypertension	Diabetes Mellitus	Smoking	Anticoagulant Agent
Case 1	97	Male	L	+	+	-	+
Case 2	74	Male	R	+	-	-	-
Case 3	81	Male	L	+	-	+	-
Case 4	91	Female	L	+	-	-	+
Case 5	69	Female	R	+	-	-	+
Case 6	64	Male	R	-	+	+	+
Case 7	82	Male	L	+	+	+	+
Case 8	84	Male	L	+	-	-	-
Case 9	93	Female	L	+	-	-	-
Case 10	78	Female	L	+	-	-	-
Case 11	77	Male	R	-	-	-	-
Case 12	93	Male	R	-	-	+	-
Case 13	82	Male	L	+	-	+	+
Case 14	76	Female	R	+	-	-	-
Case 15	90	Female	L	+	-	-	-
Case 16	76	Male	R	+	-	+	-

RPE = retinal pigment epithelium; VEGF = vascular endothelial growth factor; nAMD = neovascular age-related macular degeneration L = left; R = right; + = yes; - = no.

**Table 2:** Ophthalmic clinical characteristics of 16 patients who developed RPE tears after intravitreal anti-VEGF drug injections for nAMD.

	Lesion	PED Type	PED Height before Treatment (µm)	PED Area (mm^2^)	Cleft	Anti-VEGF Agent	Injections before RPE Tear (*n*)	Time from First Treatment to RPE Tear (days)	Tear Grade	Fovea Involvement
Case 1	AMD occult	Fibrovascular	820	8.22	-	Aflibercept	3	98	3	-
Case 2	AMD occult	Serous vascular	820	20.18	+	None	0	6	3	-
Case 3	AMD occult	Fibrovascular	550	unmeasurable	+	Ranibizumab →aflibercept	17→1	1235	4	+
Case 4	AMD occult	Hemorrhagic + Fibrovascular	450	11.68	+	Aflibercept	1	35	3	-
Case 5	AMD occult	Hemorrhagic	405	32.3	+	Aflibercept	11	658	4	+
Case 6	AMD occult	Fibrovascular	609	33.23	+	Aflibercept	1	15	4	+
Case 7	AMD classic	Fibrovascular	720	21.07	-	Ranibizumab	2	41	3	-
Case 8	AMD occult	Fibrovascular	582	26.45	+	Aflibercept	7	217	4	+
Case 9	AMD occult	Fibrovascular	483	19.31	+	Aflibercept	2	63	4	+
Case 10	AMD occult	Fibrovascular	905	18.14	-	Aflibercept	1	28	4	+
Case 11	PCV	Hemorrhagic	424	Unmeasurable	-	Ranibizumab	5	434	3	-
Case 12	AMD occult	Hemorrhagic	660	Unmeasurable	+	Aflibercept	7	325	4	+
Case 13	AMD occult	Hemorrhagic	681	Unmeasurable	+	Aflibercept	10	490	4	+
Case 14	AMD occult	Fibrovascular	274	20.26	-	Ranibizumab	1	21	3	-
Case 15	AMD occult	Fibrovascular	738	18.86	+	Ranibizumab	3	102	3	-
Case 16	AMD occult	Serous vascular	730	21.85	-	Aflibercept	8	448	4	+

RPE = retinal pigment epithelium; VEGF = vascular endothelial growth factor; nAMD = neovascular age-related macular degeneration; PED = pigment epithelial detachment; AMD = age-related macular degeneration; + = yes; - = no.

## Data Availability

Data sharing is not applicable to this article. Since the data of all cases that caused RPE tear are disclosed in the text, there is no further data to be disclosed.
